# Horizontal and vertical growth of *S. cerevisiae* metabolic network

**DOI:** 10.1186/1471-2148-11-301

**Published:** 2011-10-14

**Authors:** Luigi Grassi, Anna Tramontano

**Affiliations:** 1 Physics Department, Sapienza University of Rome, Piazzale Aldo Moro, 5 I-00185 Roma, Italy; 2 Istituto Pasteur - FondazioneCenci Bolognetti, Sapienza University of Rome, Piazzale Aldo Moro, 5 I-00185 Roma, Italy

## Abstract

**Background:**

The growth and development of a biological organism is reflected by its metabolic network, the evolution of which relies on the essential gene duplication mechanism. There are two current views about the evolution of metabolic networks. The retrograde model hypothesizes that a pathway evolves by recruiting novel enzymes in a direction opposite to the metabolic flow. The patchwork model is instead based on the assumption that the evolution is based on the exploitation of broad-specificity enzymes capable of catalysing a variety of metabolic reactions.

**Results:**

We analysed a well-studied unicellular eukaryotic organism, *S. cerevisiae*, and studied the effect of the removal of paralogous gene products on its metabolic network. Our results, obtained using different paralog and network definitions, show that, after an initial period when gene duplication was indeed instrumental in expanding the metabolic space, the latter reached an equilibrium and subsequent gene duplications were used as a source of more specialized enzymes rather than as a source of novel reactions. We also show that the switch between the two evolutionary strategies in *S. cerevisiae *can be dated to about 350 million years ago.

**Conclusions:**

Our data, obtained through a novel analysis methodology, strongly supports the hypothesis that the patchwork model better explains the more recent evolution of the *S. cerevisiae *metabolic network. Interestingly, the effects of a patchwork strategy acting before the Euascomycete-Hemiascomycete divergence are still detectable today.

## Background

Metabolism defines the reactions that provide energy and constituents for cells and organisms. It includes all the features related to the growth and the development of a living organism. The modular units of the metabolism are the metabolic pathways, sets of chemical reactions by which a metabolite is transformed into another through a series of steps catalysed by enzymes. The evolutionary history of enzymes necessarily reflects the evolution of the organism they belong to and therefore constitutes an interesting subject of study.

The metabolic networks provide a global view of the metabolic pathways of an organism and can be represented in two alternative ways. In enzyme-centric networks, proteins are defined as nodes, connected by the metabolites they process, whilst metabolite-centric networks assign the metabolites to nodes and the processing enzymes to edges.

Although metabolic networks are, without doubt, the most studied biological networks, which models are appropriate for explaining their evolutionary history is still a matter of debate. Two main models have been presented in the literature for their evolution: the retrograde model and the patchwork model (for a recent review see [1]). In the retrograde model, pathways evolve backwards from a key metabolite [2]. According to this model, enzymes are recruited in a direction opposite to the metabolic flow in an environment assumed to be rich in metabolites that become initial key metabolites or intermediates. This abundance of initial or intermediate metabolites is difficult to reconcile with the network evolution that must occur when organic molecules are depleted from the environment. The patchwork model was formulated with the aim of overcoming this conceptual limitation [3,4]. This alternative model hypothesizes an important role for broad-specificity enzymes (capable of catalysing a variety of metabolic reactions) in forging the evolution of metabolic pathways. These enzymes, thanks to their broad substrate specificities, might be involved in many metabolic pathways for the synthesis of the same or similar metabolites. In such context, gene duplications would bring a selective advantage to the pathways: an increased level of the enzyme will indeed cause an increase of the key metabolites. Finally, the specialization of the different pathways could occur as a result of specialization of paralogous enzymes.

Previous analyses showed that paralogous enzymes tend be closer in the metabolic network than expected by chance [5,6]. Although this result can be interpreted as a confirmation of the retrograde model, other studies [7,8] showed that homologous enzymes are found in different pathways more often than in the same pathway, in accordance with the patchwork model. The current way to reconcile these different findings is to postulate complementarity of the two evolutionary models, which are commonly accepted to be non mutually exclusive [9].

In this study we propose a new approach for the study of the evolution of metabolic networks based on the evaluation of the effects of the gene duplication on a given network. As test case, we selected the *S. cerevisiae *metabolic network.

*S. cerevisiae *is a well studied eukaryotic mono-cellular organism for which much information about metabolic reactions has accumulated and is reported in thousands of papers as well as included in publicly available databases. Several studies also confirmed that *S. cerevisiae *has experienced a whole genome duplication (WGD) around 100 million years ago [10-12]. Moreover, the results of a careful study [13] traced the evolutionary history of protein-coding genes from seventeen genomes of Ascomycota fungi and identified orthologs and paralogs at different periods of duplication.

The results of the analysis reported here show that the *S. cerevisiae *metabolic network has evolved in a discontinuous manner: the first growing moves increased the number of metabolites. Subsequently, all newly added enzymes did not increase the number of metabolites, but rather specialized for specific cellular compartments and/or cell cycle phase or, alternatively, were included in existing complexes. The datation of the duplications also indicates that this shift in network evolution occurred before the Euascomycete-Hemiascomycete divergence, estimated to have happened around 350 million years ago [14]. This discontinuity in the growing modality, together with other results discussed here, strongly supports the hypothesis that the patchwork model played a main role in forging the evolution of the *S. cerevisiae *metabolic network.

## Results

### Evolutionary perspective

In this study we propose an alternative perspective for the study of the evolutionary history of metabolic networks. Our primary interest is focused on the novelty of the enzymes progressively added to the network. The metabolic space can be defined as the set of all the metabolites present in a metabolic network. The addition of a new enzyme to the metabolic network can have essentially two consequences: i) the creation of a new functionality (i.e. a new metabolic reaction); ii) the duplication of a functionality already present in the network.

The innovation brought by enzymes progressively added to a metabolic network is related with the evolutionary debate between neutralism and selectionism, elegantly reconciled by the analysis of Wagner [15,16].

**Figure 1:**
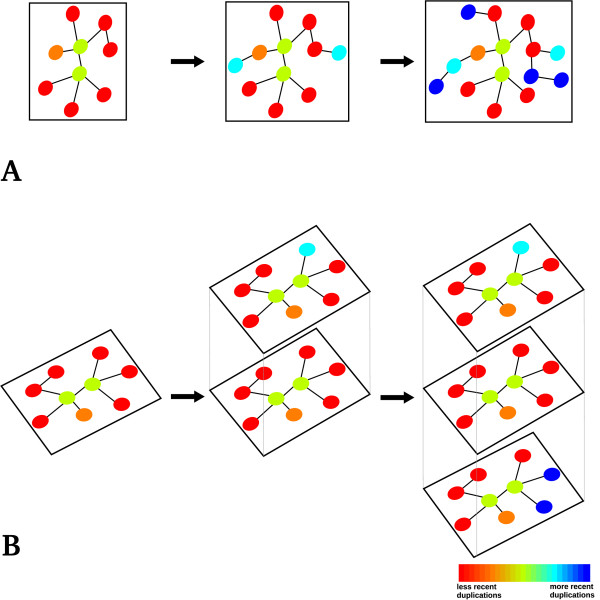
**Network growth models**. The duplication of proteins involved in metabolic reactions results in growth of the metabolic network. Paralogous pairs that process different metabolites increase the number of nodes and lead to a horizontal expansion of the network, as depicted in part A. Paralogs catalysing the same reactions, for example in different conditions, would result in a more specialized expression of the proteins and lead to a vertical growth of the metabolic network with the number of nodes remaining constant (part B). The colour scale goes from red (less recent duplications) to blue (more recent duplications).

The addition of a new enzyme that processes new metabolites would lead to an expansion of the metabolic space, reflected into the horizontal growth of the metabolic network (Figure 1A). Alternatively the vertical growth of the metabolic network is realised each time an enzyme able to process metabolites already present in the metabolic space is added to the network (Figure 1B). We studied the effects of the progressive addition of paralogous enzymes to the metabolic network by evaluating their effects on the growth of the metabolic space.

### Network analysis

In order to evaluate the contribution of paralogous enzymes to the metabolic network of *S. cerevisiae *we collapsed the network by maintaining only one member of each paralogous family (collapsed network). Next, we compared the properties of the metabolic space of the original network with those of 1000 collapsed networks (i.e. networks where the paralogous enzymes to be removed were randomly selected, see Methods). The analysis was performed in six replicates starting from two independent metabolic network databases and three paralogous family definitions. The metabolite-centric networks were reconstructed by linking products and reagents through the processing enzymes (see Methods). The two initial networks were the Yeast Biochemical Pathways (YBP), a manually curated and corrected version of the Metacyc database [17,18] available at the Saccharomyces Genome Database (SGD) [19,20] and a network reconstructed according to the reactions present in the KEGG database [21,22]. The paralogous families were defined according to three strategies. The first was based on the comparison of all protein pairs. All pairs of proteins were aligned using the EMBOSS *needle *program [23] and those with more than 30% sequence identity were connected in the paralogy network (see Methods). The connected components of this network defined the protein families and, consequently, members of the same family were identified as paralogous proteins. The analysis was repeated also using the paralogous families retrieved from ENSEMBL compara [24] and from the study of Wapinski *et al*. [13].

As first test, we compared the metabolic space of the empirical network with those of the collapsed networks obtained upon removal of paralogous proteins. The aim of this comparison was to understand whether gene duplication contributes to a vertical or a horizontal growth of the metabolic network. Clearly, the metabolic space of the collapsed network would be smaller than that of the empirical one only if paralogous enzymes would add new metabolites to the network. This would not be the case, for example, if paralogous enzymes would be mostly responsible for catalysing the same reaction in different compartments or in different conditions.

Collapsing the paralogs according to global sequence alignment led to the removal of 311 and 198 edges for the KEGG and YBP networks, respectively. When the ENSEMBL definition of paralogs was used, the corresponding figures were 265 and 113 edges. The Wapinski definition of paralogs led to the removal of 152 and 178 edges for the KEGG and YBP networks, respectively.

**Figure 2:**
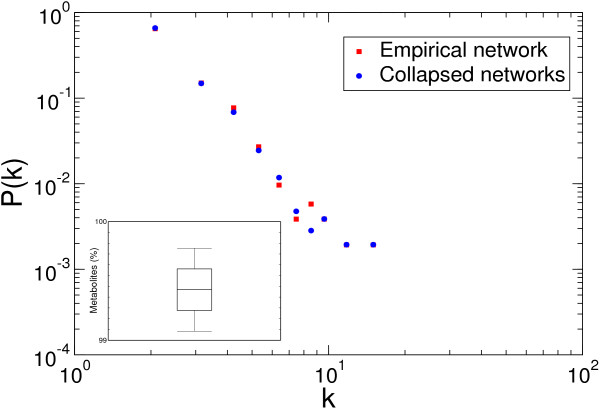
**Effects of paralog removal on the metabolic network**. The main figure reports the connectivity distributions P(k) for metabolites in the empirical network (red squares) and in 1000 collapsed networks (blue dots). The two distributions are very similar. In the inset a boxplot reporting the fraction of metabolites of the empirical network present in the 1000 collapsed networks is shown. Dashed lines indicate the mean, the upper and lower box margins correspond to the standard deviation (SD), and whiskers indicate two SD.

We consistently obtained the same result for all replicates: the collapsed networks includes the large majority of the metabolites of the empirical network. As it can be seen in the inset of Figure 2, which reports the percentage of metabolites in the YBP network collapsed according to the alignment paralog definition with respect to the empirical one, between 99% and 100% of the metabolites are preserved (see Additional file 1, Figure S1 for data obtained using different metabolic network and paralogy definitions).

The effect of the removal of a node from a network is strictly dependent on its connectivity or degree, i.e. on the number of links between it and other nodes.

Figure 2 shows that the metabolites in the empirical and 1000 collapsed networks have equivalent connectivity distributions. This result is independent of the choice of the paralogous families and of the reference metabolic network (see Additional file 1, Figure S2) and indicates that paralogous proteins have similar connectivity.

### Functional overlap of paralogous proteins

In the metabolic networks under examination, enzymes are represented as edges. In order to define the functional properties of each enzyme we listed all pairs of metabolites (nodes) it connects. Each reaction was considered only once, regardless of its reversibility, e.g. the reaction A ⇌ B was listed as AB, but not as BA (see Metabolic Network Reconstruction section in Methods). We compared the paralogous pairs present in the metabolic network in order to quantify their functional overlap by calculating the relative Dice coefficient [25]. Given two paralogous enzymes E1 and E2, the Dice_M _coefficient is defined as twice the number of overlapping metabolite pairs (intersection) divided by the total number of metabolite pairs (union): Dice_M _= Dice = 2(X1∩X2)/(X1∪X2) where X1 and X2 are the metabolite pairs of E1 and E2. This coefficient is equal to zero for enzymes with no functional overlap, whilst it is equal to one for totally functionally overlapping enzymes.

**Figure 3:**
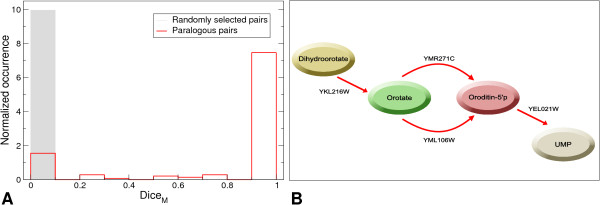
**Functional overlap of paralogous pairs**. (A) Dice_M _coefficient for all the paralogous pairs (red bars). Grey bars refer to 10^6 ^randomly selected pairs. (B) Scheme of the pathway section including two isozymes YMR271C (URA10) e YML106W (URA5) sharing 75% amino acid similarity and responsible for the same reaction, the conversion of orotate into orotidine-5'-phosphate.

Figure 3A shows the normalized distributions of the Dice_M _coefficient for the paralogous pairs (defined through the paralogy alignment network) of enzymes in the YBP network and for one million of randomly assorted pairs. Most of the paralogous pairs (~80%) are made by enzymes performing exactly the same reaction and the remaining fraction is formed by an about equal number of paralogous enzymes with no functional overlap (~10%) and of paralogous enzymes with a partial functional overlap (~10%). This result, confirmed in all replicates (see Additional file 1, Figure S3), indicates that most enzymes that can be identified as paralogs have contributed to the vertical growth of the *S. cerevisiae *metabolic network, whilst only a small fraction contributed to its horizontal growth.

Additional file 2 reports all the paralogous pairs analyzed in this study. For each pair we also calculated the Pearson correlation coefficient between their expression levels using the data from the SGD's Expression Connection tool (http://www.yeastgenome.org/cgi-bin/expression/expressionConnection.pl).

An interesting and well-studied example of paralogous enzymes with total functional overlap is reported in figure 3B. The isozymes YMR271C (URA 10) and YML106W (URA5) are WGD paralogs [11] with very similar amino acid sequences (72% identity and 84% similarity). They both catalyze the conversion of orotate to orotidine-5'-monophosphate. URA5 accounts for almost 80% of the orotate phosphoribosyltransferase activity found in yeast cells (major isozyme) and URA10 is responsible for the remaining 20% (minor isozyme) [26,27]. Interestingly, we found their expression patterns to be mildly anti-correlated in accordance with the results of Zhang *et al*., according to whom the URA5 transcription is down-regulated by dimethyl sulfoxide and the URA10 expression is up-regulated under the same conditions [28]. The WGD paralogs YDL131W (LYS21) and YDL182W (LYS20) also constitute a case of isozymes with totally overlapping function. They have almost identical amino acid sequences (87% identity and 90% similarity) and catalyze the condensation of acetyl-CoA and alpha-ketoglutarate to form homocitrate in the lysine biosynthesis pathway. A recent study [29] showed that in condition of growth on ethanol, homocitrate is mainly synthesized through LYS21, while under fermentative metabolism, LYS21 and LYS20, play redundant roles.

### Paralogous proteins catalyzing different reactions

Although the vast majority of paralogous pairs are made by isozymes with totally overlapping functions there is a small fraction (~10%) of paralogs catalyzing different reactions. This interesting minority constitutes the few horizontal growing moves of the *S. cerevisiae *metabolic network, and we describe them in more detail here.

Table 1 reports ten pairs of the enzymes identified as paralogs by at least two paralogy assignments with no functional overlap in at least one metabolic network. A first examination of these pairs immediately highlights the enzymatic similarity of such proteins. Indeed one pair is made by enzymes having exactly the same EC number, for five pairs there is a partial overlap (one of the two enzymes has the EC number without the last digit) and four pairs have the EC numbers differing only in their last digit. This indicates that these paralogous pairs, although defined as divergent by the network analysis, are very similar from an enzymatic point of view, their only difference being at the level of the substrates they process. YBR149W (ARA1) is an arabinose dehydrogenase that catalyzes the oxidation of D-arabinose, L-xylose, L-fucose and L-galactose in the presence of NADP+; YHR104W (GRE3) its paralog is an aldose reductase that reduces the cytotoxic compound methylglyoxal (MG) to (R)-lactaldehyde, although the purified GRE3 can also catalyze the reduction of xylose into xylitol. In YBP these two proteins are mapped in different pathways: ARA1 in the dehydro-D-arabinono-1,4-lactone biosynthesis and GRE3 in the xylose metabolism. YBR149W is not present in the KEGG reaction database and therefore its Dice_M _coefficient relative to the KEGG network cannot be computed. The YLR044C (PDC1), YGR087C (PDC6) and YLR134W (PDC5) isozymes all have pyruvate decarboxylase activity with PDC1 acting as the major decarboxylase [30]. They are all paralogs of YDR380W (ARO10), a phenylpyruvate decarboxylase that catalyzes the decarboxylation of phenylpyruvate to phenylacetaldehyde [31]. In KEGG, PDC1, PDC5 and PDC6 are all mapped in the "Glycolysis/Gluconeogenesis" pathway, whilst their paralog with no functional overlap ARO10 is in the "Phenylalanine metabolism" pathway. In YBP the enzymes have a partial functional overlap, with a Dice_M _coefficient equal to 0.53. All four proteins perform the same reaction in the "isoleucine degradation", "phenylalanine degradation" and "tryptophan degradation" pathways, but ARO10 is the only one mapped in the "leucine degradation" pathway and PDC1, PDC5 and PDC6 are also exclusively mapped to the "valine degradation", "acetoin biosynthesis II", "butanediol biosynthesis" and "glucose fermentation" pathways.

**Table 1:** Paralogous pairs with divergent functions

Paralogous pairs		Paralogy definition			**Dice**_**M **_**coefficient**		Correlation of the expression levels	
**Enzyme 1**	**Enzyme 2**	**Need**	**Ens**	**Wap**	**YBP**	**KEGG**	**Pearson coeff**.	**P value**

YBR149W	YHR104W	Y	Y	N	0	N/A	0.68	2.2^-16^

YDL080C	YDR380W	Y	Y	Y	0.67	0	0.08	0.033

YDL246C	YLR070C	Y	Y	Y	N/A	0	N/A	N/A

YDR147W	YLR133W	Y	Y	N	0	0.5	0.09	0.02

YDR380W	YGR087C	Y	Y	Y	0.54	0	-0.07	0.065

YDR380W	YLR044C	Y	Y	Y	0.54	0	0.08	0.047

YDR380W	YLR134W	Y	Y	Y	0.54	0	0.01	0.76

YDR399W	YJR133W	Y	Y	Y	0	N/A	0.25	7.6^-11^

YHR123W	YNL130C	Y	Y	Y	0	0	0.50	2.2^-16^

YJR159W	YLR070C	Y	Y	Y	N/A	0	0.38	2.2^-16^

Only pairs identified as paralogs by at least by two paralogy definitions (Need: sequence identity above 30%; Ens: derived from the Ensembl database; Wap: listed by Wapinski *et al*. [13] ) and with a Dice_M _coefficient equal to 1 in at least one metabolic network are shown. The complete set of data is shown in Additional file 2.

Some studies indeed showed that ARO10 and the PDC1, PDC5 and PDC6 isozymes share some substrates but also process specific substrates [31,32].

We investigated whether the growth of the metabolic network occurs in a continuous fashion. In other words, we asked whether the different modalities of the metabolic network growth happened in different evolutionary periods. To answer this question we investigated the duplication age of the paralogous enzymes.

Following what was reported by Wapinski *et. al *[13], we divided the paralogous pairs in four groups: i) pre-WGD Euascomycetes paralogous pairs, the most ancient duplications shared by all species of the Euascomycetes class; ii) pre-WGD Hemiascomycetes paralogous pairs, including all duplications before the WGD excluding the Hemiascomycetes class; iii) WGD paralogous pairs; iv) post-WGD paralogous pairs.

Six of the eight pairs with Dice_M _coefficient equal to zero (obtained using the YBP network definition) belong to the pre-WGD Euascomycetes group (hypergeometric test P value = 0.0007). A further confirmation is given by the analysis of the network derived by KEGG, where six of the nine pairs with Dice_M _coefficient equal to zero belong to the pre-WGD Euascomycetes group (hypergeometric test P value = 0.0005).

This result indicates that almost all paralogous pairs with no functional overlap derive from very ancient duplications, dated by Wapinski *et al*. [13] before the Euascomycete-Hemiascomycete divergence, estimated to have occurred about 350 million years ago [14].

Paralogous enzymes with no functional overlap generally belong to different pathways while the opposite is true for enzymes catalysing the same reaction. For the enzyme pairs shown in Table 1, we calculated their Dice_P _coefficient (see Methods). Most of the pairs show a Dice_P _coefficient different from 1 indicating that they tend to exclusively belong to a single pathway (further information are available in Additional file 3).

Another confirmation of this finding derives from the calculation of the mean shortest paths between all the metabolites (reagents and products) connected by the pairs of enzymes shown in Table 1 (see also Additional file 3). According to this measure, a hypothetical pair of enzymes connecting contiguous metabolites (i.e. the products of the reaction catalysed by one enzyme are the reagents of the reaction catalysed by the other) has a mean shortest path equal or close to 1. The mean shortest path for the network derived from YBP is 7.7 ± 3.3 and 6.8 ± 1.8 for the KEGG network. This indicates that the few horizontal growing moves tend to add enzymes in distant points of the *S. cerevisiae *metabolic network.

## Discussion

Gene duplication is generally accepted as a very important source of raw material for functional innovation [33-36]. In *S. cerevisiae*, many studies [37-40] pointed out the key role of gene duplication in the evolution of the regulatory networks, particularly through the loss, gain and rewiring of regulatory interactions occurring after the duplication. Our study aims at clarifying the effective contribution of gene duplication in the evolution of metabolic networks. This theme was treated by many other authors in the attempt of identifying the best model able to explain the evolution of metabolic networks. A study by Tsoka and Ouzounis [41] reports that enzymes from the same family are distributed among different pathways. Similarly, Teichmann *et al*. [8] found that homologous enzymes are twice as likely to be found in different pathways than in the same pathway. These pioneering studies interestingly converge on assigning a high likelihood to the patchwork model, nevertheless they have some limitations: both analyse *E. coli*, a prokaryotic organism in which the widespread phenomenon of lateral gene transfer, particularly affecting proteins involved in metabolism [42], can compromise the reliability of the homology inference. Furthermore both studies do not analyse the metabolism from a network viewpoint and this can be misleading since the metabolic reactions in cells, even if mapped in different pathways, may be close in the network. Subsequent studies analysed the metabolism from a network perspective. Alves *et al*. [5] and Díaz-Mejía *et al*. [6] analysed the metabolic networks of 12 species and of *E. coli*, respectively, reaching similar conclusions: homologous enzymes tend to be closer in the network than expected by chance and, at the same time display a strong chemical similarity (essentially catalyse very similar reactions). However, these results cannot be used to discriminate between the retrograde or patchwork model. In the first scenario, the neighbourhood of duplicated enzymes can be interpreted as a proof of a stepwise growth of the metabolic network consequent to a stepwise addition of enzymes. On the other hand the enzymatic similarity of paralogous enzymes can also be interpreted as supporting the patchwork model, according to which broad specific enzymes tend to catalyse chemically similar reactions even when acting on different types of substrates [43]. These studies mapped the paralogous enzymes in an enzyme-centric metabolic network, which has the problem that a short distance between two totally overlapping enzymes cannot be distinguished from a signal resulting from contiguous enzymes. For this reason, here we selected to approach the problem using a metabolite-centric reconstruction of the metabolic network, which is more informative. For example, let us consider two totally overlapping paralogs E1 and E2, processing the same reaction from metabolite A to metabolite B and let us assume that both A and B are next processed to give a different metabolite C. In an enzyme-centric network the two enzymes would have a distance equal to 2, while in our approach, enzymes E1 and E2 would have a Dice_M _coefficient equal to 1 and would be considered as totally overlapping paralogs.

Our results strongly support the conclusion that the vast majority of paralogous pairs nowadays detectable in *S. cerevisiae *are similar to our E1 and E2 hypothetical enzymes and do not contribute to the horizontal expansion of the metabolic network.

This conclusion is robust and independent on the definition of metabolic network and/or on the method used for paralogy assignment.

In fact, the metabolic space remains mostly unchanged upon removal of paralogous enzymes and of their connected metabolites, consistently with previous studies demonstrating a strong conservation of the biochemical function in *S. cerevisiae *duplicated genes [13,44,45].

Furthermore, Gash *et al*. demonstrated that *S. cerevisiae *isozymes show different transcriptional regulation with specific members of isozyme families responding to environmental stress [46] and the study of Ihmels *et al*. [47] elegantly showed that the isozyme multiplicity is a strategy adopted by *S. cerevisiae *to permit differential regulation of reactions shared by different processes. Such strategy provides the possibility to have independent control on the associated reactions in response to pathway-specific requirements.

Although most of paralogous enzymes are isozymes with completely overlapping functions, there is a small fraction (~10% of pairs) of paralogs catalysing totally non-overlapping reactions. The characterization of these divergent paralogs is useful to uncover the evolutionary process behind the definition of the metabolic network of *S. cerevisiae*. Interestingly, they are almost always derived from very ancient duplications, dated by Wapinski *et al*. [13] before the Euascomycete-Hemiascomycete divergence (about 350 million years ago). This result indicates a discontinuity in the growth modalities of the *S. cerevisiae *metabolic network, with the metabolic space remaining unaltered or almost unaltered for 350 million years.

Interestingly, this growth strategy is in accordance with the theoretical prediction that the appearance of enzymes occurs in bursts rather than by phyletic gradualism proposed by Schütte *et al*. [48].

This may be the result of reaching a network equilibrium, after which all the growing moves add already existing enzymatic reactions rather than creating novel functionalities. The differentiation of broad-specificity enzymes hypothesized by the patchwork model could have been the event that contributed to reach such equilibrium, after which all enzymes added to the network were already specialized in performing one specific reaction.

Although one could expect that, also in the retrograde model, the equilibrium state could have been reached, for example, once all the possible biochemical reactions had been explored, in this second scenario, one would expect to see signs of the retrograde model. The last horizontal growing moves should result in the duplicated enzymes being close in the metabolic network, but this is not the case: almost all the paralogs with partial or no functional overlap are mapped to different pathways and the mean distance of the metabolites they process is not as short as one could expect according to the retrograde model.

Our results also suggest that the methodology used here is a powerful and straightforward way for studying the effect of paralogous enzymes on the metabolism of an organism and unravel its evolutionary strategy.

## Conclusions

The purpose of our analysis was to evaluate the contribution of the paralogous enzymes to the growth of the metabolic network. In order to do this we used a simple strategy: the comparison of the empirical metabolic network with collapsed networks, obtained through the random removal of paralogous enzymes. This approach, applied to the specific case of *S. cerevisiae*, gave interesting results. We obtain the same results regardless of the paralog and network definitions used: the metabolic network of *S. cerevisiae *grew discontinuously. The few moves able to expand the metabolic space are in large part due to gene duplications dated before the Euascomycete-Hemiascomycete divergence. Almost all subsequent duplications gave raise to enzymes performing the same reaction, but specialized for different cellular localizations or differently regulated. This result suggests that the patchwork model is more compatible than the retrograde model with the growth modality of the metabolic network of *S. cerevisiae*. A further confirmation of this is given by the finding that all the paralogs with partial or no functional overlap are far away in the metabolic network. In conclusion, the described method was found to be useful for studying the contribution of paralogous enzymes to the metabolism of *S. cerevisiae *and can be applied to the evolutionary study of the metabolism of other organisms.

## Methods

### Metabolic Networks Reconstruction

All the analyses performed in this study relied on two metabolic networks: the YBP network and the KEGG network. Both are metabolite-centric networks, reconstructed by connecting substrate and products through their processing enzymes. The networks are represented by directed graphs where nodes denote compounds and edges denote proteins. The directionality of the edges reflects the direction of the reaction. Reactions catalyzed by protein complexes were represented including the same reaction for each member of the complex. Highly connected hubs can be a source of noise [49], therefore we restricted our analysis to the main reactants in both networks, excluding the currency metabolites from the analysis.

The YBP network was derived by SGD [19,20] (http://downloads.yeastgenome.org/curation/literature/archive/yeastcyc14.0.tar.201009.gz).

We referred to SGD Pathway Tools version 14.0. The file pathways.dat contains all the details for each reaction present in the database (enzymes, substrates, products, and directionality). We also used to the files protcplxs.col and enzrxns.dat. The first is a detailed list of the protein complexes and of all their components, whilst the other describes all enzyme features (gene name, modality of action of dimer/monomer and the catalyzed reaction). Only reactants classified as "primaries" were taken into account.

The KEGG network was derived from the KEGG PATHWAY Database [21,22]. The reaction file (version 04/03/2011) contains all the information about the reactions (substrate/product and reversibility). Only the RPAIRS (reactant pairs) classified by KEGG as "main" pairs were taken into account. The enzyme-reaction associations were retrieved by the file sce01100.xml (version 04/05/2011).

The names of compounds and glycans were also retrieved from the compound file and the glycan file derived from the KEGG LIGAND database.

### Paralogy detection

All our analyses were performed using three paralogy definitions. A paralogy network was defined by considering all the proteins of *S. cerevisiae *as nodes. All predicted ORFs sequences in the *S. cerevisiae *genome were downloaded from Ensembl release 59 (http://aug2010.archive.ensembl.org/) [24]. The amino acid sequence identity was computed using *needle*, a global alignment algorithm included in the EMBOSS suite of programs [23]. Pairs with more than 30% sequence identity were considered paralogs. The other two paralogy assignments were derived from ENSEMBL compara, release 59 (http://aug2010.archive.ensembl.org/) [24] and from the results of the analysis reported by Wapinski *et al*. [13].

### Network analysis

Networks were analysed using the Cytoscape freeware (version 2.8.0) [50]. The calculation of the connectivity was made using an in-house developed Perl script.

All properties of the empirical networks were compared with those of randomly collapsed networks. For each paralogous family formed by at least two enzymes present in the metabolic network, one enzyme, randomly chosen, was kept while all the others (together with their connected nodes) were removed. The ShortestPath finder plugin for Cytoscape (http://csresources.sourceforge.net/ShortestPath/) was used to calculate the shortest path length among the metabolites.

### Dice coefficient

The functional overlap between paralogs was measured by the Dice coefficient [25], using two enzyme properties.

For two E1 and E2 enzymes, the coefficient was defined as (Dice_M_) twice the number of shared metabolite pairs over the sum of metabolite pairs processed by the enzymes and (Dice_P_) as twice the number of common pathways divided by the number of pathways in which the enzymes are involved:

Dice=2(X1∩X2)(X1∪X2)

Where X1 and X2 are the number of metabolite pairs or pathways, respectively.

The reversibility of the reactions was not taken into account. The order of the two metabolites was substrate → product (KEGG) or Left → Right (YBP). Pathway definitions were obtained from KEGG and YBP Metacyc.

### Comparison of the expression of paralogous enzymes

The similarity of the transcriptional regulation of paralogous enzymes was assessed through their Pearson's correlation coefficient, calculated using the data available at the SGD expression connection tool (http://www.yeastgenome.org/cgi-bin/expression/expressionConnection.pl). This includes 29 datasets obtained in different experiments. We joined all the data present in the pre-clustered files (in log2 space) in a global expression table. From this table we removed the columns (experiments) with more than 5% missing genes and the lines (genes) with more than 5% missing experiments. The filtered table, containing 5294 genes and 658 experiments, was used to calculate the Pearson's correlation coefficient between paralogous pairs.

## Abbreviations

SGD: Saccharomyces Genome Database; YBP: Yeast Biochemical Pathways; WGD: whole genome duplication.

## Authors' contributions

AT and LG designed the project and wrote the paper. LG performed the analyses. All authors read and approved the final manuscript.

## Supplementary Material

Additional file 1 **Supplementary Figures**. Supplementary figures reporting the data obtained using alternative definitions of the metabolic network and/or paralogous pairs. Click here for file

Additional file 2 **Supplementary Table 1**. Detailed list of all the studied paralogous pairs. For each pair the paralogy assignment by all the three methods, the Dice_M _coefficient relative to both networks and the Pearson correlation coefficient of their expression levels with the relative P values are reported. Click here for file

Additional file 3 **Supplementary Table 2**. Paralogous pairs with no or partial functional overlap. The table reports the Dice_P _coefficient and the shortest path length for the pairs listed in Table 1. Click here for file
