# Supplementary material for: Horizontal and vertical growth of S. cerevisiae metabolic network
Source: BMC Evol Biol. 2011 Oct 14;11:301. doi: 10.1186/1471-2148-11-301 (PMC3216907; doi:10.1186/1471-2148-11-301)
Supplement: Additional file 1 — Supplementary Figures. Supplementary figures reporting the data obtained using alternative definitions of the metabolic network and/or paralogous pairs. [file 1471-2148-11-301-S1.PDF]

Supplementary Figures

Horizontal and vertical growth of the S. cerevisiae metabolic network

Luigi Grassi,[1]
and Anna Tamontano[1,2,†]

[1]Physics Department, Sapienza University of Rome, Piazzale Aldo Moro, 5
I-00185 Roma, Italy.

[2]Istituto Pasteur – Fondazione Cenci Bolognetti, Sapienza University of Rome,
Piazzale Aldo Moro, 5 I-00185 Roma, Italy.

† corresponding author, email: anna.tramontano@uniroma1.it

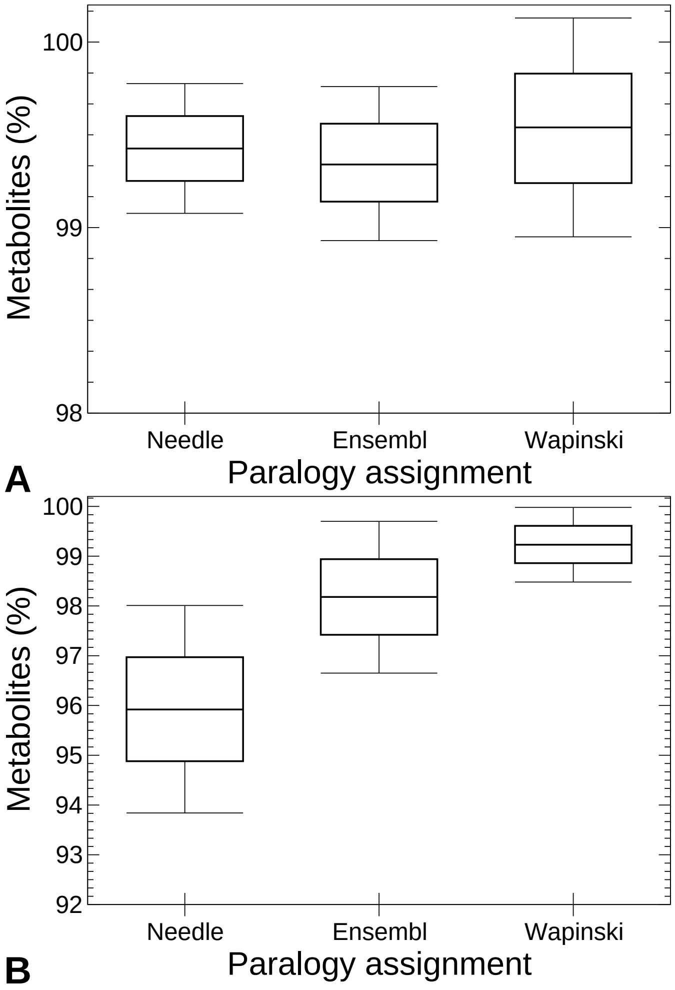

Figure S1: **Metabolic space of paralogous enzymes.** The fraction of metabolites of the empirical network present in the 1000 collapsed networks is reported as boxplots: dashed lines indicate the mean, the upper and lower box margins correspond to the standard deviation (SD), whiskers indicate two SD. Parts A and B refer to the results relative to YBP and KEGG networks, respectively.

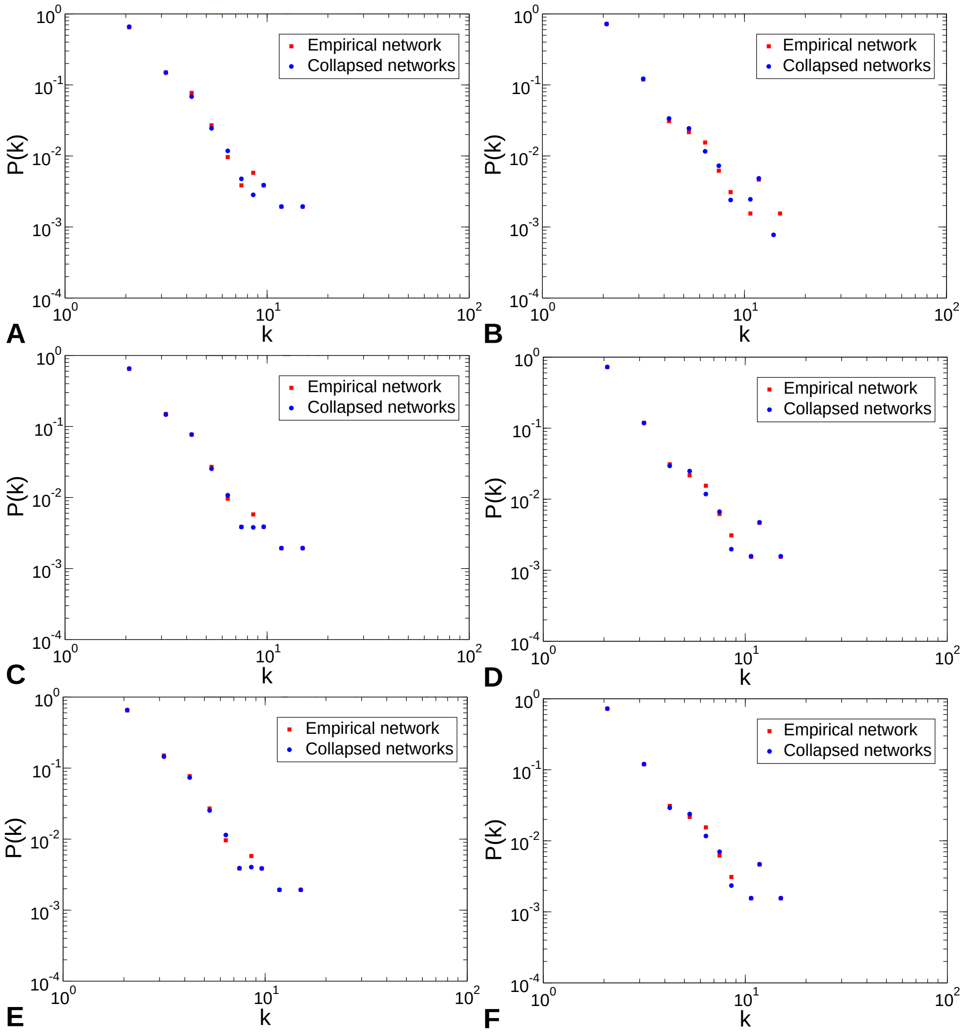

Figure S2: **Connectivity distributions of empirical and collapsed networks.** Connectivity distributions P(k) for metabolites in the empirical network (red squares) and 1000 collapsed networks (blue dots). Parts A, C and E refer to the YBP network and parts B, D and F to the KEGG network. The paralog definitions are protein alignment (A and B), Ensembl compara (C and D) and the results of Wapinski *et al.* [1] (E and F).

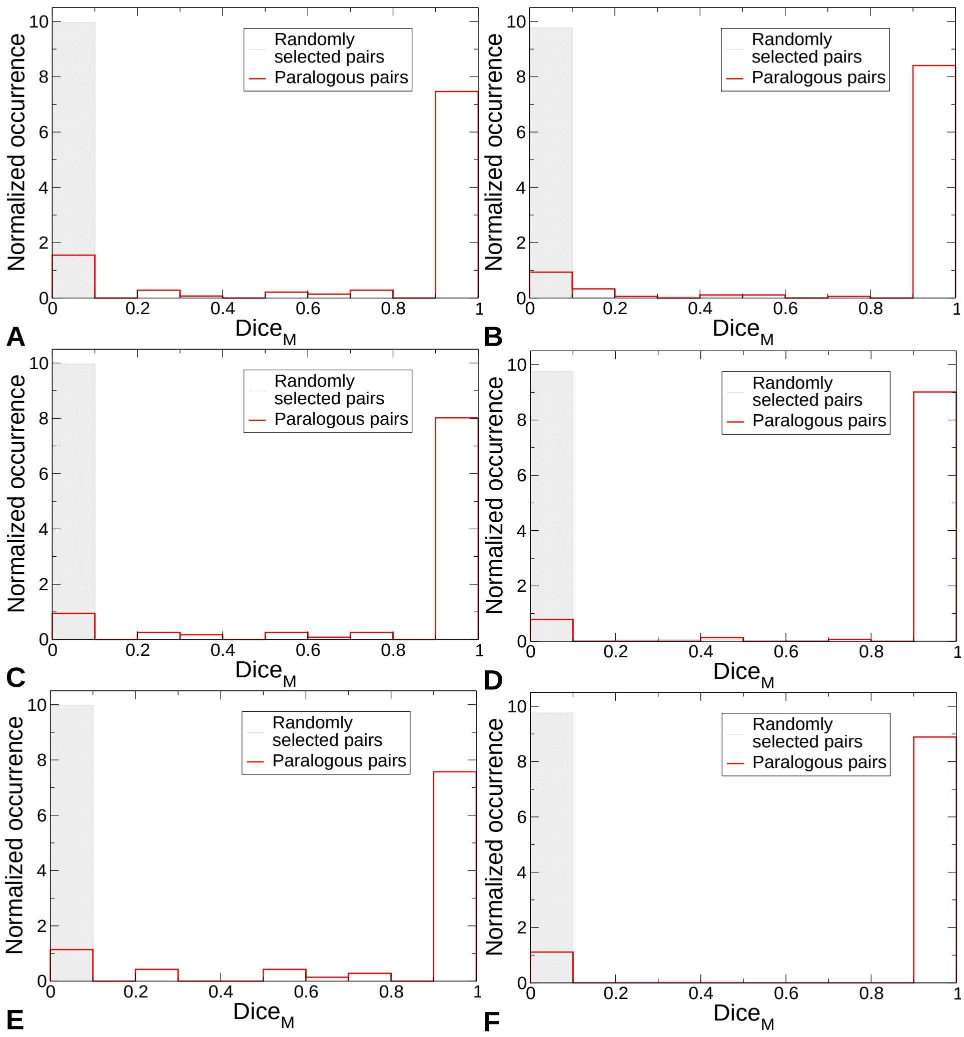

Figure S3: **Functional overlap of paralogous pairs.** Dice$_M$ coefficient for the paralogous pairs (red bars). Grey bars refer to 10^6 randomly selected pairs. Parts A, C and E refer to the YBP network and parts B, D and F to the KEGG network. The paralog definitions are protein alignment (A and B), Ensembl compara (C and D) and the results of Wapinski *et al.* [1] (E and F).

References

[1] Wapinski I, Pfeffer A, Friedman N, Regev A: **Natural history and evolutionary principles of gene duplication in fungi**. *Nature* 2007, **449**(7158):54–61.
